# Unraveling the influence of task designs and intrinsic motivation in effort-based decision-making

**DOI:** 10.3758/s13421-025-01745-6

**Published:** 2025-07-08

**Authors:** Alyssa Randez, Sébastien Hélie

**Affiliations:** Department of Psychological Sciences, Purdue University, West Lafayette, IN 47907 USA

**Keywords:** Cognitive effort-based decision-making, Task preferences, Demand preferences, Capability, Incentives

## Abstract

Cognitive effort-based decision-making typically focuses on how much effort a person chooses to invest in a given action. Past experiments follow the assumption that monetary means and cognitive demands influence effort investment. However, motivation can influence behaviors beyond these factors, leaving open the question as to how individual tasks can motivate effort. Using two experiments, this study investigated how different elements of task designs can contribute to preferences that are related to the costs of performing an action and the rewards of monetary incentives. The results of this study suggest that preferences for an option can be influenced by various task-related factors such as the cognitive components required to complete a task (e.g., motor- or memory-related), how demanding the options are relative to each other (as determined by the number of elements to be manipulated in the task), and how much reward is available after task completion. In some cases, task designs could explain decision-making tendencies better than monetary reward or demand levels. These findings have significant implications for understanding how intrinsic motivation affects preferences based on the requirements of a task that can be unrelated to ability.

**Supplementary Information:**

The online version contains supplementary material available at 10.3758/s13421-025-01745-6.

## Introduction

Intrinsic motivation is often acknowledged for driving individual differences in how and when someone chooses to perform an effort action (e.g., Oudeyer et al., 2016, Oudeyer, 2018; Ryan & Deci, 2000). These motivations are said to differ in many ways from extrinsic ones in that people who are considered intrinsically motivated find reward sources internally, with the reward coming from the experience of increasing one’s knowledge and skills in a task or knowledge set (Oudeyer et al., 2016), to relieving boredom (Agrawal et al., 2022) and motivating long-term extrinsic rewards by tackling more achievable actions that may not immediately lead to these rewards. However, while the importance of these internal motivations appears to be necessary in terms of evolution (Berlyne, 1960) and learning in general (Aubret et al., 2023; Oudeyer, 2018), less is known about how highly intrinsically motivated people experience these internal rewards.

Theories of effort investment in decision-making suggest that individual differences in seeking out cognitively challenging tasks can be explained by differences in cognitive abilities (Agrawal et al., 2022; Hardy et al., 2019; Westbrook & Braver, 2015). Resource-oriented models assume that decision-makers use similar strategies when making effort-based decisions, and that these decisions are primarily driven by differences in the demand costs they experience rather than differences in motivation (e.g., Botvinick et al., 2009; Rangel et al., 2008; Westbrook et al., 2013). In this view, these theories complement economical models of decision-making which state that ability can explain the variance in effort investment because higher-performing individuals have more cognitive “currency” to spend (Kool et al., 2010; Shah & Oppenheimer, 2008). Specifically, when an action is too demanding, the action places the decision-maker in a state where they cannot succeed, whereas an action that is not demanding enough does not help the decision-maker learn (Agrawal et al., 2022; Hardy et al., 2014; Hardy III et al., 2019; Oudeyer, 2018). Ability-cost theories suggest that actions are motivating because they place the decision-maker in an optimal state of engagement. State-oriented and resource-oriented models explain intrinsic motivation reflecting individual differences in ability and demand costs. This explanation offers a testable prediction that people will pursue actions related to their ability to perform that action.

Other theories of effort investment suggest that actions are selected with a higher focus on the external rewards. These theories propose that differences in reward sensitivity, or how likely someone is to be motivated by an external reward such as money or points, can further predict how likely a person is to perform an effortful action (Sandra & Otto, 2018). Under this viewpoint, external and internal rewards motivate people differently in that some are more sensitive to external reward like money while others are more sensitive to internal rewards such as learning in a way that complements other theories of intrinsic motivation (Cacioppo & Petty, 1982; Ryan & Deci, 2000).These theories propose that, rather than differences in demand costs, differences in cognitive effort-based decision-making are more strongly related to the source of the reward: individuals who are intrinsically motivated are more sensitive to internal rewards than those who appear more motivated by external rewards (Oudeyer, 2018; Ryan & Deci, 2000; Sandra & Otto, 2018).

In an attempt to disentangle the role of cognitive ability and demand costs, Randez and Hélie (2024) investigated individual differences in demand preferences by tailoring each demand level to the person. The results suggest that controlling for differences in demand costs did not eliminate individual differences in demand preferences: some participants still indicated a preference for the high demand task. This is contrary to the prediction that people will generally prefer options that are more cost efficient, and questions support for the idea that people who prefer high demanding tasks only show this preference because that option may be easier for them when compared to others (Botvinick et al., 2009; Kool et al., 2010). These results indicate that demand costs may not have been driving these choices. Instead, an exploratory analysis suggested that the type of task used across the experiments may have played an important role in influencing participants’ decisions.

However, comparing the influence of task designs can be challenging when trying to identify which features of a task can be considered attractive (Awasthi & Pratt, 1990; Richardson et al., 2001). The bulk of empirical research on task attractiveness has primarily been conducted in industrial environments, where productivity and motivation can be incentive- and performance-based (e.g., Bailey & Fessler, 2011; Bonner & Sprinkle, 2002; Fessler, 2003; Rafinda et al., 2021; Robinson & Farkas, 2021). Cognitive research has a lot of empirical evidence studying two components that often appear in various tasks: motor and memory skills (e.g., Hélie & Pizlo, 2022; Hinze et al., 2009; Pizlo & Stefano, 2013). Relating these empirical findings to more varied tasks, memory and motor movement appear to be useful to accomplish complex skills such as learning a musical instrument or gaining expertise (Richardson et al., 2001). While focusing on these two components can be limited in generalizing to the vast collection of tasks, using tasks that feature one or both of these factors can perhaps provide insight into effort-based decision-making. Specifically, comparing performance and decisions that are primarily motor-based or memory-based can help provide support for these tasks being demand-oriented (i.e., do people prefer tasks that they are better at?) or more intrinsic to the task (i.e., they have a strong motor or memory-related preference but may not be related to their performance).

With this aim, the current article investigates whether individual differences in effort-based decision-making that cannot be fully explained by demand and reward factors are instead related to different types of tasks being offered. Similar to Randez & Hélie (2024), this involves examining the influence of factors such as cognitive ability, demand levels, and reward sensitivity on task preferences. The results of these two experiments suggest that there may be intrinsic motivation related to the task designs which support learning-oriented frameworks (Oudeyer, 2018; Ryan & Deci, 2000): People may seek out actions they find enjoyable to perform for their own sake.

## Experiment 1: Demand-related decisions

Experiment 1 investigated whether individual differences in capability could predict choices among different task types. While answering why someone would choose an activity such as playing a musical instrument or video game over another one would be challenging, this study instead attempted to compare how preferences are influenced by changes in different factors of similar tasks. With this aim, this study limited task designs to two dimensions: the number of task elements and the type of cognitive components required. As a result, six options were offered, including two demand levels of three cognitive tasks designed to be as visually similar as possible (described in the next section) to investigate whether participants’ performance predicted preference patterns among these task options. If participants primarily based preferences for options on ability, then their choices among the six options should be predictable based on performance. If, however, choices are influenced by other task aspects, then participants should demonstrate preferences for specific tasks that may be separable from their performance on that task.

### Methods

#### Participants

One hundred undergraduates from Purdue University were granted course credits for participation. All participants were enrolled in an undergraduate Introduction to Psychology course and were at least 18 years old. Participants provided written consent, and the Purdue University Institutional Review Board approved the experiment. Two participants were excluded due to computer malfunctioning issues, leaving the total sample size at 98. A priori power analysis for a 2 (demand level) × 3 (task type) within-subject ANOVA calculated the need for at least 83 participants to achieve a power of.80, assuming similar group differences among task accuracy found in pilot studies, with alpha at.05 and an effect size of Cohen’s *d* =.76.

#### Task options

This experiment was programmed using PsychoPy3 (Pierce et al., 2019). Stimuli were presented on a 21-in. monitor (1,920 × 1,080 resolution), and responses were recorded using a standard keyboard. Stimuli were presented across a visual angle of 14.9° with a height of 5 in. and a width of 3 in.

The tasks presented were designed to be as visually similar in presentation as possible, with the main difference being the cognitive requirements of the participants. For the type of cognitive components, this experiment used two one-component tasks that primarily required a motor or a memory component. A two-component task was also created, which required both a memory and a motor component, referred to as the “hybrid task.” Pilot testing was done to select the number of elements in each task to achieve a relatively similar target performance across participants (60% accuracy for the high-demand options and 80% for the low-demand options). Elements here were the number of squares within each task component that the participants had to memorize or respond to.

##### Same-different “memory” task

**Fig. 1:**
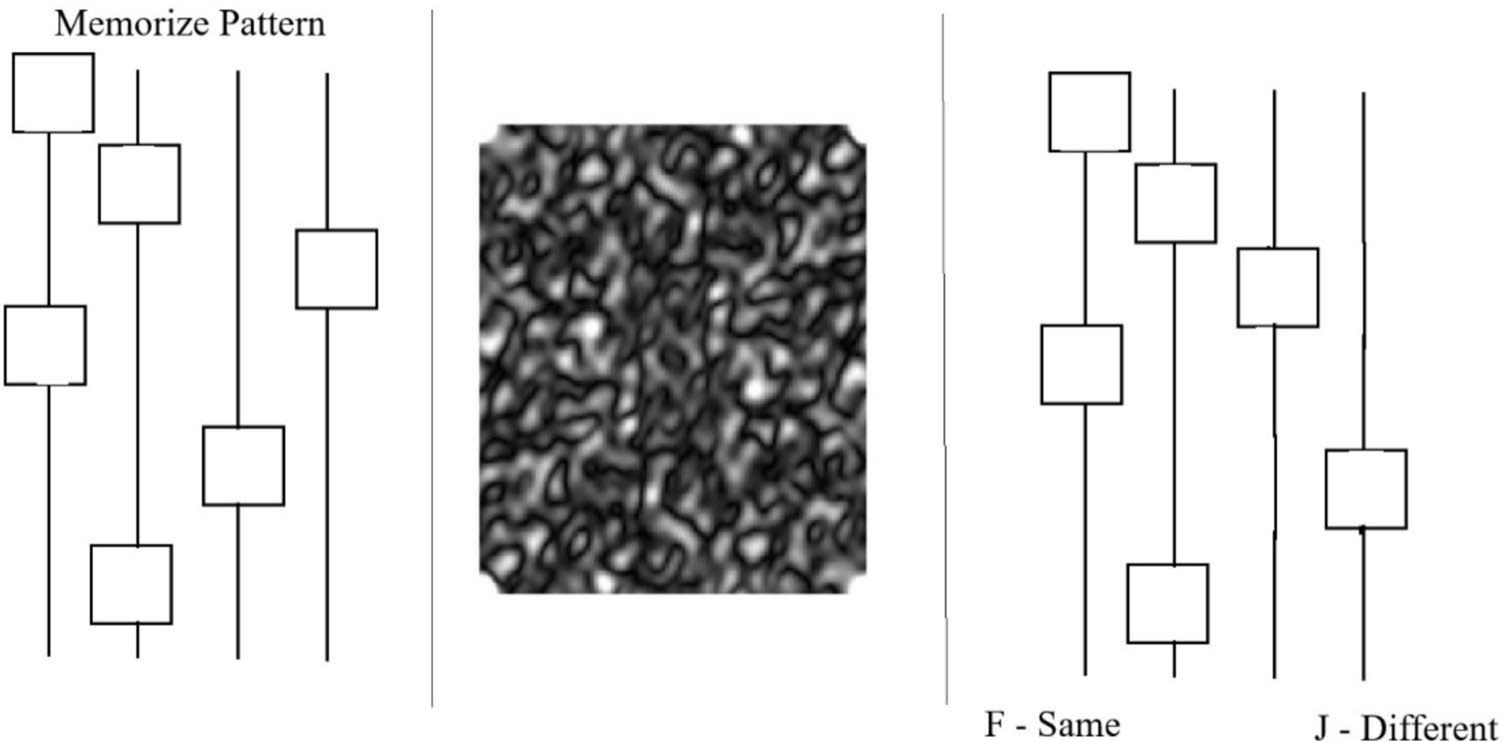
Example trial of the memory task. This figure shows the three parts of a trial in which the first pattern is presented (**left**), followed by a masking screen (**middle**), and then a second pattern is presented (**right**)

This task was designed to require memory cognitive components. Participants saw four vertical black lines on a gray screen with a pattern of squares on the lines. The number of squares for the low-demand option was six squares, and 11 squares for the high-demand option. Participants were given 2 s to memorize the pattern, after which they saw a masking screen covering the area of the four lines (6 in. × 6.5 in.) for 1.5 s before they were shown the four lines with the same number of squares on the lines (Fig. 1). Participants were instructed to press “F” if this second pattern matched the first pattern and “J” if not. There was a 50% chance of the second pattern being the same and a 50% chance that the second pattern swapped positions of the two squares from the first pattern. Feedback was given near the top of the screen as either “correct” or “incorrect” and remained on the screen during the intertrial interval (ITI) of 1.5 s.

##### “Motor”-perception task

**Fig. 2:**
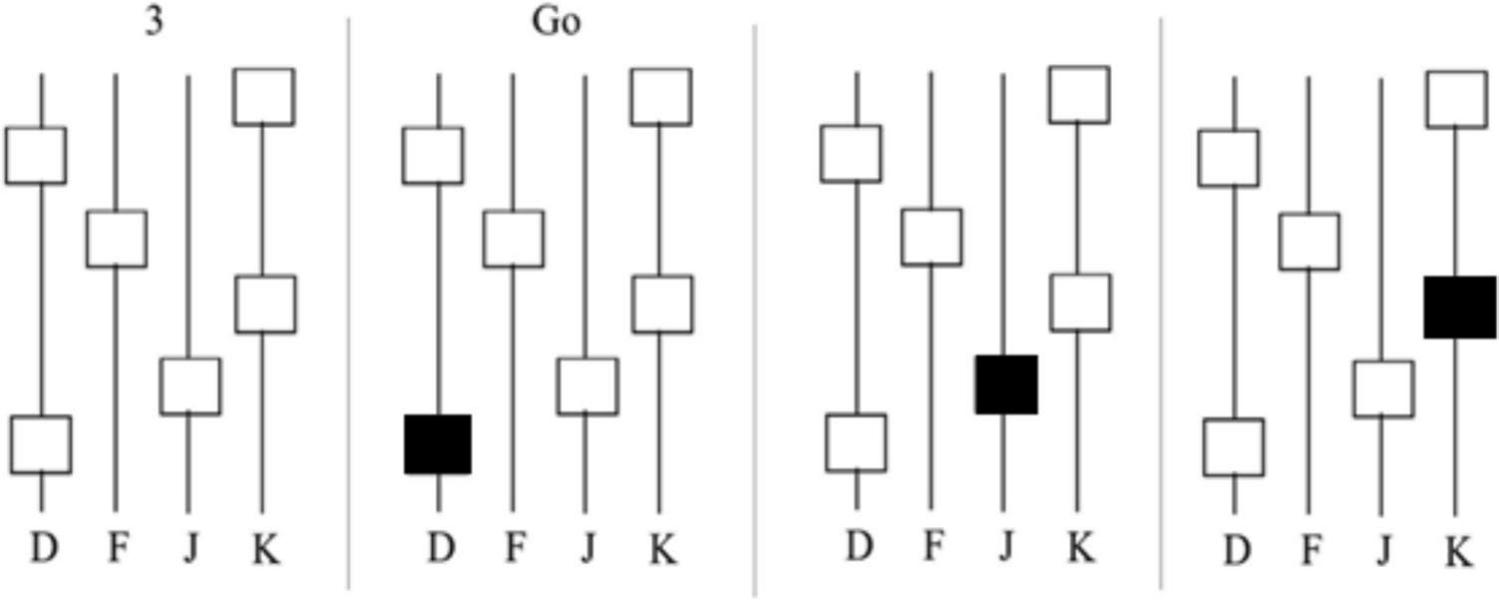
Example trial of the Motor task. In the figure above, from left to right, participants saw a pattern of squares and a timer starting from 3 to 1. When the timer reached 1, the word “Go” appeared, and participants were instructed to strike the key that matched the letter below the green box before the next square turned green

The Motor task had a similar setup to the memory task: four vertical black lines with four squares in the low-demand option and six in the high-demand option. Below each line were the letters d, f, j, and k, respectively. Participants saw a countdown timer above the four lines starting from 3 to 1. After 1, the word “Go” appeared, and the squares turned green one at a time from the bottom to the top of the screen (Fig. 2). Only one box was green at any given moment, and the participant was instructed to press the key associated with the lit box before the next box turned green. The total duration for both high- and low-demand levels was 3 s, so high-demand trials required faster button pressing. The intertrial timing was set to 1.5 s, in which the word “correct” or “incorrect” appeared at the top of the screen until the subsequent trial began.

##### Spatial/Working Memory “Hybrid” task

**Fig. 3:**
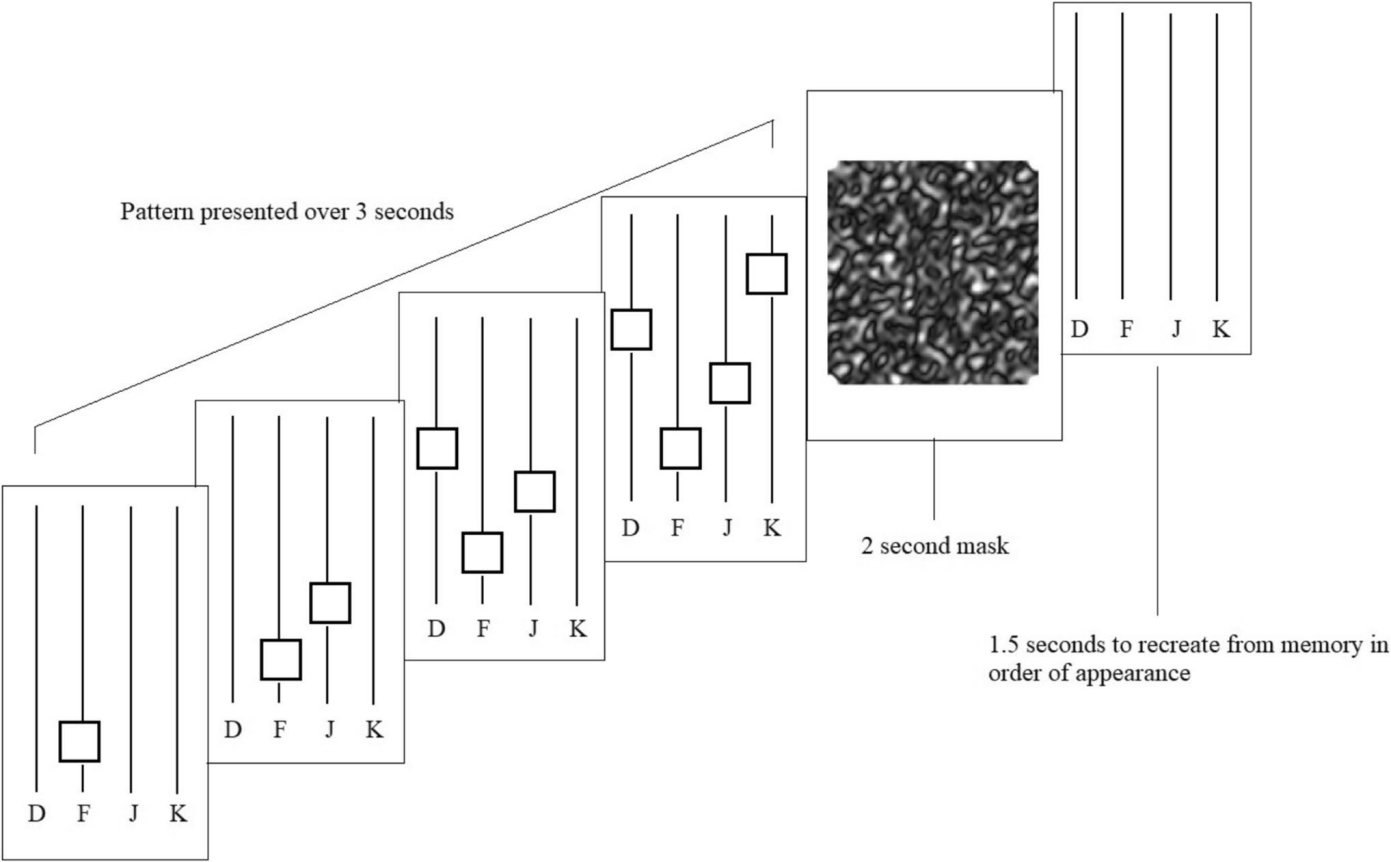
Example trial of the Spatial Memory Hybrid task*.* Participants were instructed to memorize patterns by order of appearance and recreate the patterns by memory after seeing a mask

The Hybrid task showed four lines in the center of the screen and used the same visual display setup as the previous two tasks. The number of squares in the low-demand option was four, with six in the high-demand option. Like the memory task, participants were given 3 s to memorize the pattern in the order of appearance as the squares appeared from bottom to top, followed by a masking screen for 2 s. Participants then saw four black lines, each with a letter below them (d, f, j, and k, respectively). They were expected to recreate the square pattern from memory within 1.5 s after viewing a mask for 2 s (Fig. 3). As a result, similar to the motor task, the high-demanding trial required faster button pressing.

Feedback was presented in two forms: whenever a key was pressed, the square for that keystroke appeared in green if it was correct or black if the key was incorrect, and the word “correct” or “incorrect” remained on the screen during the 1.5-s intertrial interval after the last button was pressed. If a key was incorrect, the trial continued, and the participants could choose at their discretion whether to complete the trial – once an incorrect key was pressed or trial time ran out, that trial was marked as incorrect.

#### Procedure

Tasks and demand levels were presented consecutively for all participants. For each task, the low-demand version was presented as “Task”.1 and the high-demand as “Task”.2; the order of tasks was as follows: Same-Different “Memory” task, “Motor” task, and Spatial/Working Memory “Hybrid” task. Participants were shown an example trial of each task they were to perform before performing practice trials. For the memory task, they were instructed that their accuracy was binary – either they recognized the pattern or they did not. For the motor task, they were instructed that both timing and accuracy were important. For the working memory task, they were instructed to complete the trial from bottom to top (in order of appearance) to get the trial correct. Participants performed 15 trials in each block, and this performance was used to build the demand-related models (described below). After this exposure phase, participants entered a choice phase in which each combination of task options was presented two at a time, six times each, for a total of 90 choices. Each option pair was randomized in order of presentation, with each combination being seen once before repeating option pairs. Participants were allowed to freely choose which options they wanted. Since preferences can be related to the initial exposure to a task (Bandura, 1997; Bonner & Sprinkle, 2002), the participants were not required to perform each task choice and only had to perform 13% of their choice in which they completed ten trials of the task selected. This percentage allowed for the experiment to be run within an hour to meet the credit requirements for participants.

#### Data analyses

##### Modeling criterion

This experiment utilized the QTEST software to identify qualitative patterns of choices. QTEST is a software that runs a non-parametric statistical test that allows for individual testing of decision-making hypotheses for pairwise options and then comparing these response patterns against a set of competing models (Regenwetter et al., 2014; (https://regenwetterlab.web.illinois.edu/qtest/)). Using this software, 12 models were constructed (six demand-related and six task-related models) that represent 12 different ideal patterns of responses a person would produce if their preferences were based on demand-related and task-related biases (see the Appendix in the Supplementary Material for details). This software allows us to compare these models using Bayes factor statistics (Kass & Raftery, 1995) to explore individual demand preference patterns. The following models tested whether participants showed a preference associated with their performance (Demand-related models) or whether participants showed a task preference (Task-related models).

We used two criteria when selecting the best-fit model for a participant:

1. The Bayes factor had to be over 3.12 to be considered substantial evidence (based on the Kass & Raftery, 1995, strength-of-evidence table), and
2. If two or more models fit criterion 1, the ratio between the best-fitting model and the second-best fitting model had to be above 3.12.

This would indicate that while two different models fit the data well, there was substantially more evidence for one model over the other. Based on the QTEST results, participant preferences were classified as:

1. Demand-related,
2. Task-driven, or
3. No best-fitting model.

These scenarios were considered to support the difference in motivation between demand-related (1) and task-related preferences (2) or based on another (untested) dimension (3).

##### Demand-related models

Six models were created using individual performances in the six task options. Three models were built to capture two possible motivations:

1. Those who seek to conserve effort (Demand Avoidant; DA) (Kool et al., 2010), and
2. Those who instead seek out challenges (Challenge-Seeker; CS) (Ryan & Deci, 2000).

These models mirror each other, representing similar decision strategies. Model CS represents ideal responses from challenge-seeking participants in that these participants prefer an option based on how challenging that option is, as inferred by lower performance. Model DA represents ideal responses from a cognitive miser or one who preferred options in which they performed best.

Previous research has suggested that participants use heuristics to make decisions between options – that is, when given a choice, they will not only choose options based on demand levels, but the strategies participants use to make these decisions are also generalized (e.g., Cooper-Martin, 1994; Pizlo & Stefanov, 2013). The three models in each category were broken down into the following:

1. Performances in all options were considered against each other;
2. Performance in low-demand options were primarily driving preferences, or
3. Performances in high-demand options primarily drove preferences.

##### Task models

Six models were additionally created, ranking the three tasks in all possible combinations. These models allowed for comparing task preferences that were not overtly related to the participant’s performance. For example, one may always choose the memory task, regardless of how well or poorly they performed in that task. These six models are every arrangement of possible task preference combinations. It is important to note that, unlike the demand-related models, these six task models were the same for all participants since these models were not dependent on specific abilities.

### Results

#### Accuracy

**Fig. 4:**
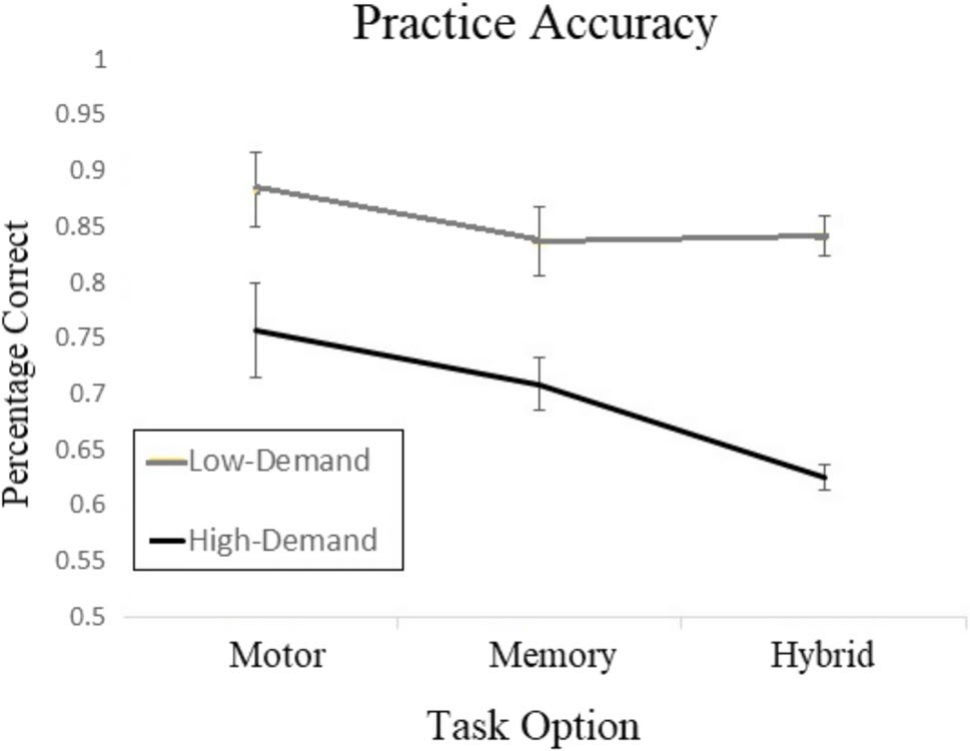
Practice accuracy averages for Experiment 1. Bars represent standard error

Practice accuracy was analyzed to investigate further the interaction between performance and preferences (Fig. 4). A 2 (high/low demand level) × 3 (task type) within-subjects ANOVA showed significant main effects of task type, *F*(2,166) = 22.459, *p* <.01, *η*_*p*_^*2*^ = 0.079, a significant main effect of levels,* F*(1,83) = 208.174, *p* <.01, *η*_*p*_^*2*^ = 0.248, and a significant interaction, *F*(1.82, 151) = 28.281, *p* <.01, *η*_*p*_^*2*^ = 0.064, between task types and levels. Differences in demand levels in each task were compared in order to ensure that the high-demand version of each task had lower accuracy than the corresponding low-demand version. For the Motor task, the high-demand level (*M* = 0.757, *SE* = 0.042) was significantly different from the low-demand level (*M* = 0.883, *SE* = 0.033), *t(*97) = 29.59, p <.01, *d* = 0.589). For the Memory task, the high-demand level (*M* = 0.709, *SE* = 0.024) was significantly different to the low-demand level (*M* = 0.837, *SE* = 0.031), *t*(97) = 35.38,* p* <.01, *d* = 0.963). Lastly, the high-demand accuracy in the Hybrid task (*M* = 0.625, *SE* = 0.011) also had lower performance than the Hybrid low-demand level accuracy (*M* = 0.842, *SE* = 0.018). This difference is numerically larger than in the other two tasks, likely explaining the interaction.

#### Decisions

**Fig. 5:**
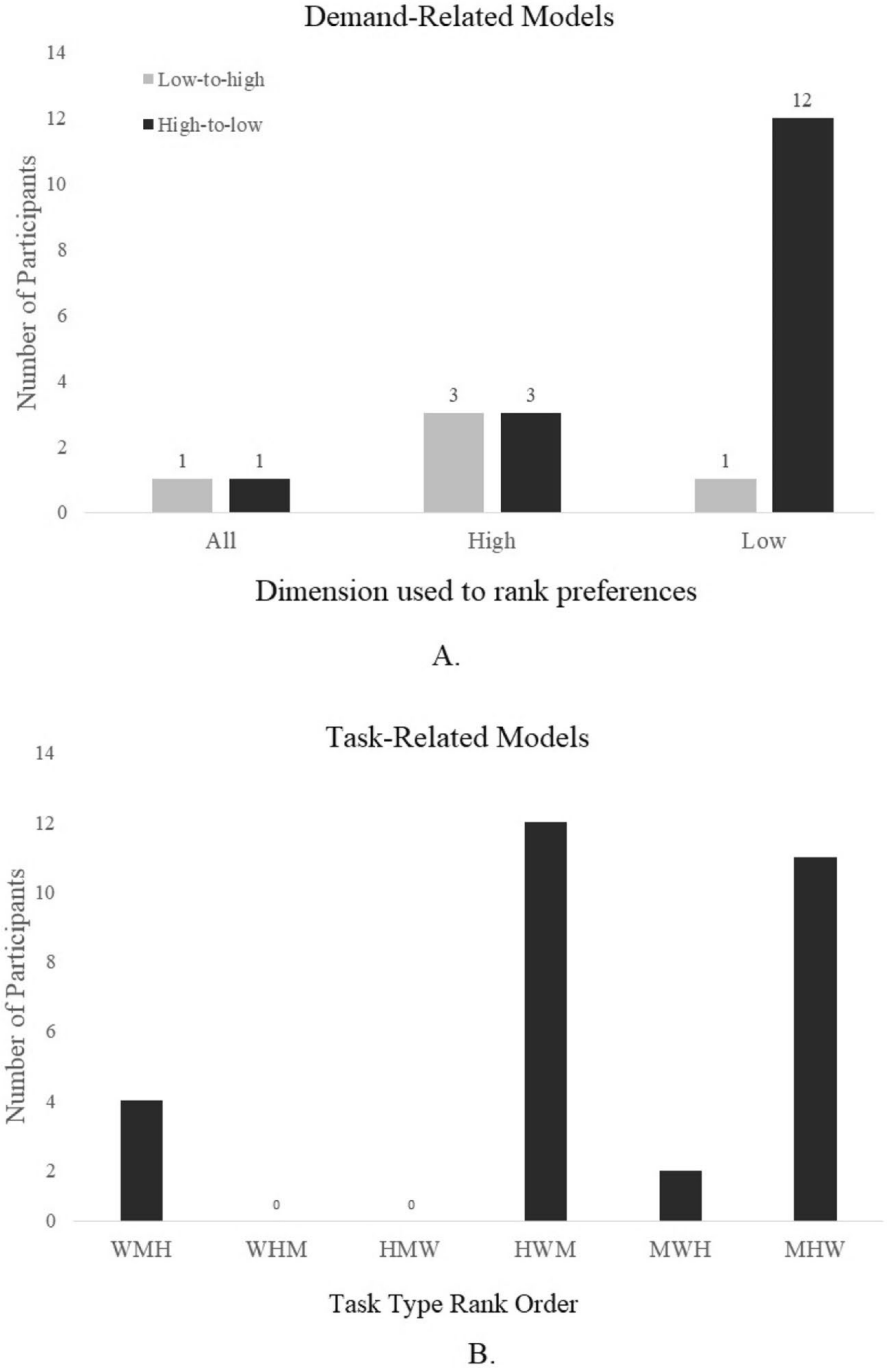
Participant best-fit models. The figure shows two types of categorizations: performance-oriented (**A**) and task-oriented (**B**). (**A**) Three dimensions of performance that participants can rank their preferences on: *All* are participants who considered each task at each demand level in relation to their performance to establish their preference ranking, *High* are those who considered their performance in the high-demand levels only to establish their preference ranking, and *Low* are those who considered their low-demand performance only to establish their preference ranking. Low-to-high represents the rank order of preferences: in this case, participants will prefer options they score higher on over those they score lower on (i.e., options that are lower demand for them). High-to-low are those who prefer options on which they score lower and thus may experience higher demand. (**B**)Models that rank-order the three tasks: for instance, W-M-H would indicate that the participant preferred the memory over the motor over the hybrid task

Demand-related and task-related models were compared. Fifty participants made choices that best fit one of these 12 models. For the demand-related models, 21 participants made choices consistent with their performance on each option during the exposure phase (Fig. 5A). Twenty-nine participants indicated that they had a rank-over preference based on task but not necessarily related to performance during their exposure phase (Fig. 5B).

To illustrate how the QTEST models allow for a more fine-grained analysis of participants’ choices, two participants were compared. One participant indicated a task-oriented preference and the second participant indicated an ability-oriented preference. Out of 72 options, the number of times a task was chosen over another was taken, and both participants showed similar rank-order preferences. The ability-oriented participant chose the hybrid task 38 times, the motor task 22 times, and the memory task 12 times when the respective tasks were offered over the other two tasks. The task-oriented participants had a similar set of choices: 47 for the Hybrid task, 18 for the motor task, and six for the memory task. Taking these proportions of choices, both participants indicate a rank-order task preference of hybrid, then motor, then memory. However, these participants differed in the order of performance on these tasks. The ability-oriented participants indicated they made decisions based on one of the high-demand dimensions models, and their performance for the tasks was as follows, in order of least to greatest: Hybrid 45%, Motor 68%, and Memory 73%. On the other hand, the task-oriented participant had the following ordered performance: Hybrid 54%, Memory 68%, and Motor 73%. This is one example of how ability can influence one participant, but its influence on another is unclear.

**Fig. 6:**
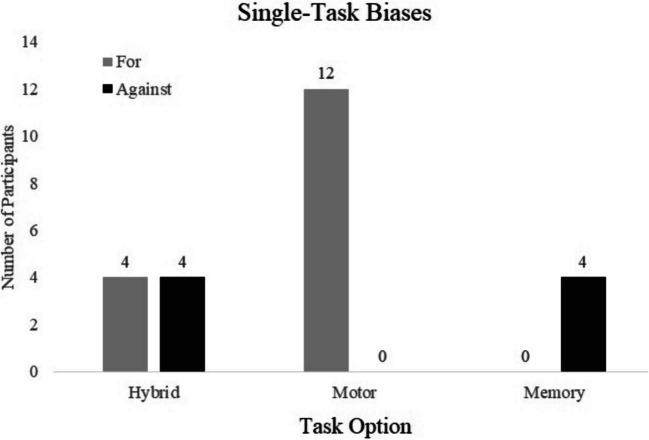
Single-task biases from the No Best Fit Group. The figure shows the number of participants who indicated a single-task preference

One limitation of using the QTEST software, however, is that task preferences needed to be ranked in an order that included all tasks (to provide a sufficient number of constraints) and did not include the possibility that preferences could be biased for or towards one task option (thus ignoring the other two). The following is a breakdown of participants who did not fit one of the 12 models but whose choices indicated that they either mostly preferred or avoided one option and were potentially agnostic toward the other two options. A Bayes factor analysis (Kass & Raftery, 1995) was conducted to isolate task preferences from random noise. As these results were not predicted, they should be considered exploratory. This analysis indicated that eight participants demonstrated a bias related to the hybrid option (four for and four against). In comparison, 12 participants indicated a preference for the motor task and four an aversion for the memory task (Fig. 6).

### Discussion

This experiment investigated how preferences were affected by task designs that varied in two forms:

1. The number of elements (low vs. high demand), and
2. Component types (motor or memory).

While some participant data were not best fit by any of the 12 models, evidence still suggested that the majority of participants’ decisions were related to specific task offerings. Only four participants produced responses that did not differ from a random model. It is also noteworthy that the lower accuracy in the hybrid task (and the larger difference between the difficulty levels) did not result in a particular aversion toward that task, with preferences similar to the other two tasks. Given that participants needed to have a fully completed trial in the memory and hybrid task, this could have led to “quitting early” in that the participant stopped investing effort once they realized they made a mistake. This could account for the lower accuracy seen in the higher demanding hybrid task. However, this may add support for the idea that decision strategies can be influenced by factors other than demand-related ones. When comparing task components, these results provided evidence suggesting that motor and memory components were favored differently depending on the individual in way that is not overtly related to that individual’s ability.

## Experiment 2: Incentive-related decisions

The results of Experiment 1 suggested that the type of task being offered can influence decisions beyond the relative demands of the task. This second experiment expands on these findings by including a reward-sensitivity factor in addition to various demand levels and task designs. Specifically, this second experiment investigated the effects of task components in monetary and demand-related contexts. Traditional paradigms comparing decisions in these incentive conditions typically raised the demand level and the monetary amount simultaneously, assuming these factors affect choices in a particular direction (away from high demand and toward high reward) (e.g., Westbrook et al., 2013). These economic paradigms suggest that participants will avoid more demanding options and seek more monetarily rewarding ones. However, one limitation of this paradigm is that it cannot answer whether people motivated away by higher demands are more motivated by monetary incentives (Westbrook et al., 2013).

Experiment 2 compared a demand-related to a reward-related condition to investigate whether preferences for low demand and high reward were influenced differently when offered two tasks that differed in design. The main research question being investigated was whether participants made similar demand-related and reward-related decisions, and whether task designs could explain these decisions. If participants primarily make economic decisions regarding cognitive resources and monetary gains, then participants who make more demand-avoidant decisions should make higher-rewarding decisions, regardless of the task being offered. If task designs influence decisions or a person is making non-economic decisions (e.g., preferring less money or higher demanding options), then a preferred task should be selected even when it is offered at a higher demand level or lower monetary reward.

The same tasks as in Experiment 1 were used to facilitate comparison. However, the structure of these tasks made it difficult to scale the difficulty of the Hybrid task to match the difficulty of the motor or memory task. Pilot studies suggest that adding a square increased the difficulty in the Hybrid task more dramatically than adding a square in either the memory or the motor task. This led to assigning the hybrid task as a *primary task* and the motor and memory tasks as *secondary tasks*. The aims of this current experiment were to compare how incentive conditions (reward or demand) influenced participants within-subjects while using the Hybrid and secondary tasks to allow for an additional task comparison. While this limited our ability to compare how participants chose among all three tasks, this design allowed for the comparison of the motor and memory tasks in a between-subjects design and the incentive conditions within participant.

### Methods

#### Participants

One hundred and forty-seven students were recruited using the Purdue University undergraduate pool. All participants were over 18 years old and enrolled into an undergraduate Introduction to Psychology course. They received three credits toward course completion and a small monetary amount (up to $5.00) based on choices made in the experiment. This study used a 2 (between task type: working memory or motor) × 2 (within adjustment: incentive or demand) repeated-measures mixed-subject design. A priori power analysis using G*Power 3.1.9.4 (Faul et al., 2007) was run for a mixed ANOVA within-between interactions to investigate task accuracy. This analysis indicated that at least 64 total participants would be needed for a power of.80 and an alpha of.05. Participants provided written consent, and the Purdue University Institutional Review Board approved the experiment. This experiment was pre-registered with the Open Science Framework (https://osf.io/23exs/).

#### Materials and procedures

This experiment was programmed using PsychoPy3 (Pierce et al., 2019). Stimuli were presented on a 21-in. monitor (1,920 × 1,080 resolution), and responses were recorded using a standard keyboard. Stimuli were presented across a visual angle of 14.9° with a height of 5 in. and a width of 3 in.

#### Task options

This experiment used the same tasks as Experiment 1.

#### Procedure

The procedure included four phases: survey, practice, reward, and demand. It was conducted over one session, with the last two phases including either a reward or a demand condition, with the order of conditions counterbalanced. The first phase consisted of a 23-question survey; 18 questions were from the Need for Cognition Scale (Cacioppo & Petty, 1982), which measures an individual’s tendency to enjoy cognitively engaging activities. The five remaining questions came from the behavioral activation system (BAS) reward-responsiveness questionnaire (Carver & White, 1994), which measures a participant’s sensitivity to reward.

**Fig. 7:**
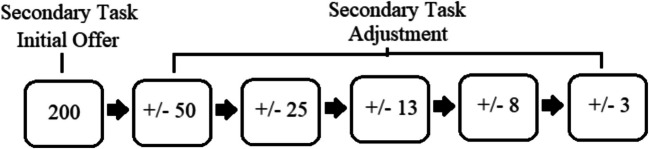
Example of a set of choices in the reward phase. The initial task was offered at 200 francs (level 6 in the demand condition) and then adjusted by the indicated amounts (by ± 1 level in the demand condition for every adjustment). This set was repeated three times before switching to the following incentive condition

The participants were randomly assigned to the memory or motor task condition. Condition assignment determined the participant’s secondary task. Participants in each condition were shown instructions for their assigned secondary task before performing 25 practice trials of their assigned secondary task. Next, participants in both conditions were shown instructions for the Hybrid task (the hybrid task is the primary task for both conditions) and performed 25 practice trials. After this, participants were asked to select between performing the hybrid task or their assigned secondary task. In the reward phase, francs were offered for each task option. In the demand condition, this number instead reflected the number of squares within the task options. To indicate a task selection, the key “C” was positioned below the left option and “N” below the right option. A set of choices included six selections, in which participants were randomly assigned to perform three to five of these selections (Fig. 7). If a block was selected to be skipped, the participant saw the screen “Recorded! Please choose again!” Otherwise, the participants performed ten trials of the task they selected. After the end of the session, participants were paid depending on their performance for the reward condition and received course credit for participating.

##### Reward phase

The reward phase used a paradigm similar to the one used in Westbrook et al. (2013). In this paradigm, one option was offered at a set number of francs while the other fluctuated. In this paradigm, the Hybrid task remained the same amount while the other option was adjusted based on the previous decision.

Currency was offered as “francs” to help disguise that the bonus compensation depended on their choices during this condition. The initial offer for each option was 200 francs each, after which the option for the secondary task adjustment began at 50 francs (increased if the Hybrid task was chosen, decreased if the secondary task was). Adjustments were chosen to closely mimic Westbrook et al. (2013) in that they decreased as follows: 50, 25, 13, 8, 4, and 1. After the sixth decision, the options reset to the initial offer states (200 francs), and adjustments began again at 50. Decision “francs” were added up to place participants in one of five payment categories, each corresponding with a dollar amount: $1.00 – less than 2,500 francs; $2.00 – between 2,501 and 3,200; $3.00 – between 3,201 and 3,900; $4.00 –between 3,901 and 4,600; and $5.00 – above 4,601.

##### Demand phase

In the demand phase, the Hybrid task with four squares was scaled to the difficulty level equal to the seven squares in the Motor and Memory tasks. Seven squares were chosen instead of six squares (as seen in Experiment 1) because this allows for the scaling design of the demand phase. Because each choice can decrease the demand level (i.e., remove a square), seven squares allows for the lowest demand level that a participant can be offered to be 1. Pilot data suggested that the difference in performance for both motor and memory tasks were similar across tasks when increasing by one square. While the Hybrid task is offered at four squares and the secondary task starts at seven squares, the choices on the screen were shown as “Hybrid: level 6” and “Motor/Memory: level 6 to try to minimize any biases that the different number of squares might have on the participant’s decisions. Past pilot studies have suggested that the Motor and Memory tasks increase in similar difficulty from adding or subtracting one square. These data also suggest that participants can achieve up to 12 squares before performance reaches a floor effect. Similar to the Reward phase, the Demand phase had six options between the Hybrid and the secondary task, and the secondary task fluctuated by the number of squares in the task performed. Every time the secondary task was selected, the number of squares increased by one square; if the Hybrid task was selected, the secondary task decreased by one square.

### Results

#### Performance

##### Practice accuracy

Half the participants saw the motor task as their secondary task, with the other half performing the memory task. A 2 (between, secondary task condition: motor vs. memory) × 2 (within**,** task type: hybrid vs. secondary task) mixed ANOVA indicated that there was a significant effect of task type, *F*(1,145) = 21.693, *p* <.04, *η*_*p*_^*2*^*.* Accuracy in the secondary task (*M =* 0.829, *SE* = 0.013) was significantly higher than in the hybrid task (*M =* 0.752, *SE* = 0.015). There was not a significant main effect of the secondary task in that participants in the motor task condition (*M* = 0.764, *SE* = 0.02) performed similarly to those in the memory task condition (*M* = 0.742, *SE* = 0.22), *F*(1,145) = 1.281, *p* =.260,* η*_*p*_^*2*^ =.006), nor a significant interaction between task type and secondary condition, *F*(1,145) = 1.686, *p* =.196,* η*_*p*_^*2*^ =.004).

##### Reward phase accuracy

**Fig. 8:**
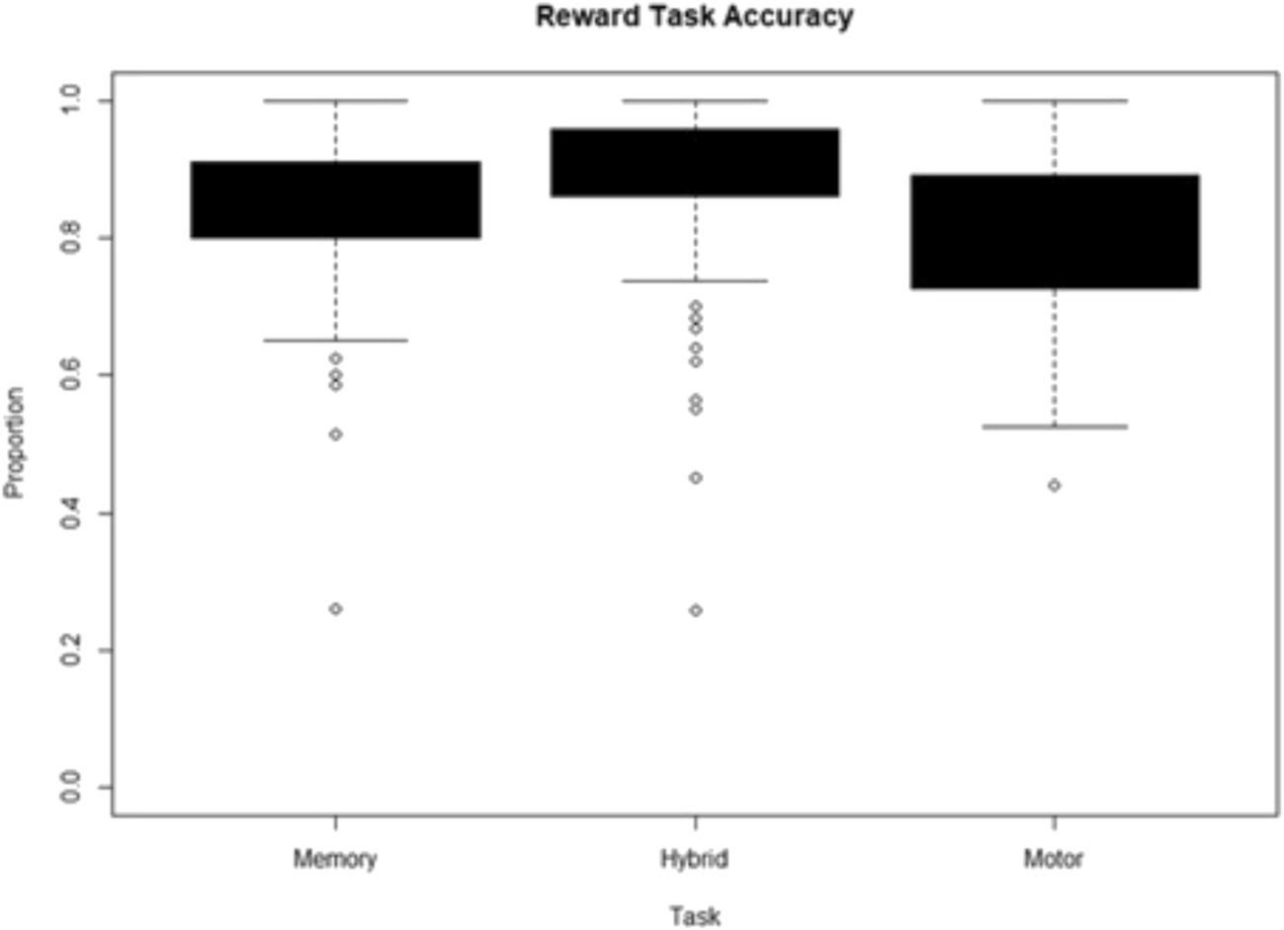
Boxplot of participants accuracy for the participants in the reward phase for each task

Participants performed only the selected tasks they chose, and 15 participants’ data were removed for not performing one of the tasks in either one or both incentive conditions. A mixed-effect 2 (selected task: Hybrid/secondary, within-subject) × 2 (secondary task condition: motor/memory, between-subject) ANOVA indicated that there was a significant effect of the selected task, *F*(1,136) = 62.310, *p* <.001, *η*_*p*_^*2*^ = 0.084, in that the secondary tasks (*M* = 0.822, *SE* = 0.010) showed lower accuracy than the Hybrid task (*M* = 0.888, *SE* = 0.009) (Fig. 8). There was no significant difference between the secondary task conditions, *F*(1,135) =.806, *p* = 0.371, *η*_*p*_^*2*^ = 0.005, nor a significant interaction, *F*(1,135) = 2.699*, p* = 0.103, *η*_*p*_^*2*^ = 0.004. A Pearson’s correlation was carried out between accuracy in tasks and the number of times that task was chosen but was not significant for either the hybrid (*r* =.-.043, *t*(140) = −0.513, *p* =.608) or the secondary tasks (*r* =.126, *t*(140) = 1.498, *p* =.136).

##### Demand phase accuracy

**Fig. 9:**
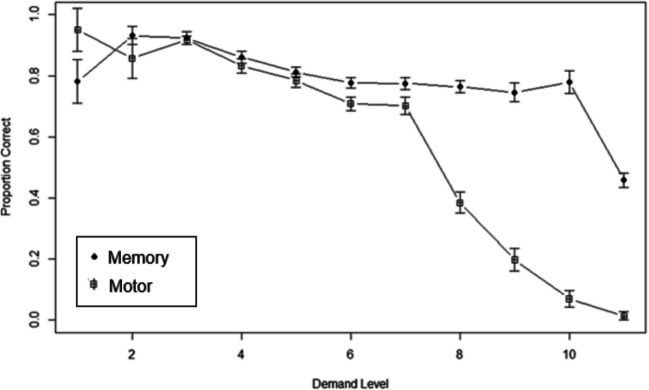
The average accuracy for the secondary (motor, memory) tasks in the demand phase for each demand level. Bars represent standard errors

The motor task demand levels ranged from one square (*n* = 2, *M* =.95, *SD* =.071) to 11 squares (*n* = 8, *M* =.013, *SD* = 0.35). The memory task demand levels ranged from one square (*n* = 12, *M* =.725, *SD* = 0.293) to 11 squares (*n* = 20, *M* =.115, *SD* =.115). However, not all levels from each task were selected, so a mixed-effect ANOVA could not be run for the demand phase due to empty cells in the different demand levels. Instead, a regression line was fit for the secondary tasks (Fig. 9). For the memory task, increases in demand offers were significantly related to decreases in accuracy, *b* = −0.027, *t*(401) = −6.939, *p* <.01, *d* = 0.327. For the motor task, increases in demand offers were also significantly related to decreases in accuracy, *b* = −0.109, *t*(388) = −18.80*, p* <.01, *d* = 0.690. These regressions were compared with each other in a Fisher’s Z correlation test, which showed a significant difference between secondary task conditions, *z* = 7.135, *p* <.001, *d* = 1255.136 (two tails). These results suggest that the demand levels for the motor task were associated with a steeper decrease in performance per additional square as the demand level increased compared to the memory task. Specifically, the motor task had about a 10% decrease in accuracy per additional square. In contrast, the memory task had about a 3% decrease in accuracy.

The hybrid task accuracy was statistically different between the two conditions, *t*(281.65) = -.2.9642*, p* <.01, with accuracy in the memory condition (*M* =.864, *SE* =.010) being higher when compared to the motor condition (*M* =.822, *SE* =.010). However, the number of choices for the Hybrid task was not statistically significant between the two task conditions *t*(272.93) = −0.521, *p* =.603). These results indicate that while performance was different, we found no evidence that this difference affected the decisions made for or against the Hybrid task.

#### Decisions

The final scores were transformed into z-scores to allow for easier comparison as lower demanding options incentive-wise may be similar to high monetary amounts and vice versa. While the demand scale was between 1 and 11 and the monetary scores were in the hundreds, the distribution of scores should follow similar logic (ending scores close to starting scores indicates task switching, while extreme scores in either direction indicates a bias). Z-scores allow us to put final demand and reward scores on a commensurable scale for direct comparison. For instance, if participants had a particularly low demand z-score, the participant chose the hybrid task when the secondary task was offered at a higher demand; likewise, a low reward z-score suggests that the participant selected the hybrid task for less money. Higher scores, on the other hand, would indicate that the secondary task was chosen when the hybrid task was offered for higher demand or less money.

Each set’s final offerings in the secondary task for the sixth decision were averaged separately in each incentive condition. These scores were transformed into z-scores using the between-subject mean and standard deviation for each set of scores. A Pearson’s correlation between z-scores revealed a significant correlation (*r* = -.472, *p* <.001, *t* = −6.3435). Participants with a higher final score in the reward condition typically had a lower demand final score. This result has two interpretations: In looking at incentive conditions only, the interpretation is that participants who are more willing to perform at higher demand levels are also less motivated by monetary means. The second interpretation is that these people instead had a preference for the Hybrid condition. This interpretation would be because their choices for the Hybrid task were consistent in both phases (i.e., they chose the Hybrid task for lower reward offerings or for higher demand levels).

**Fig. 10:**
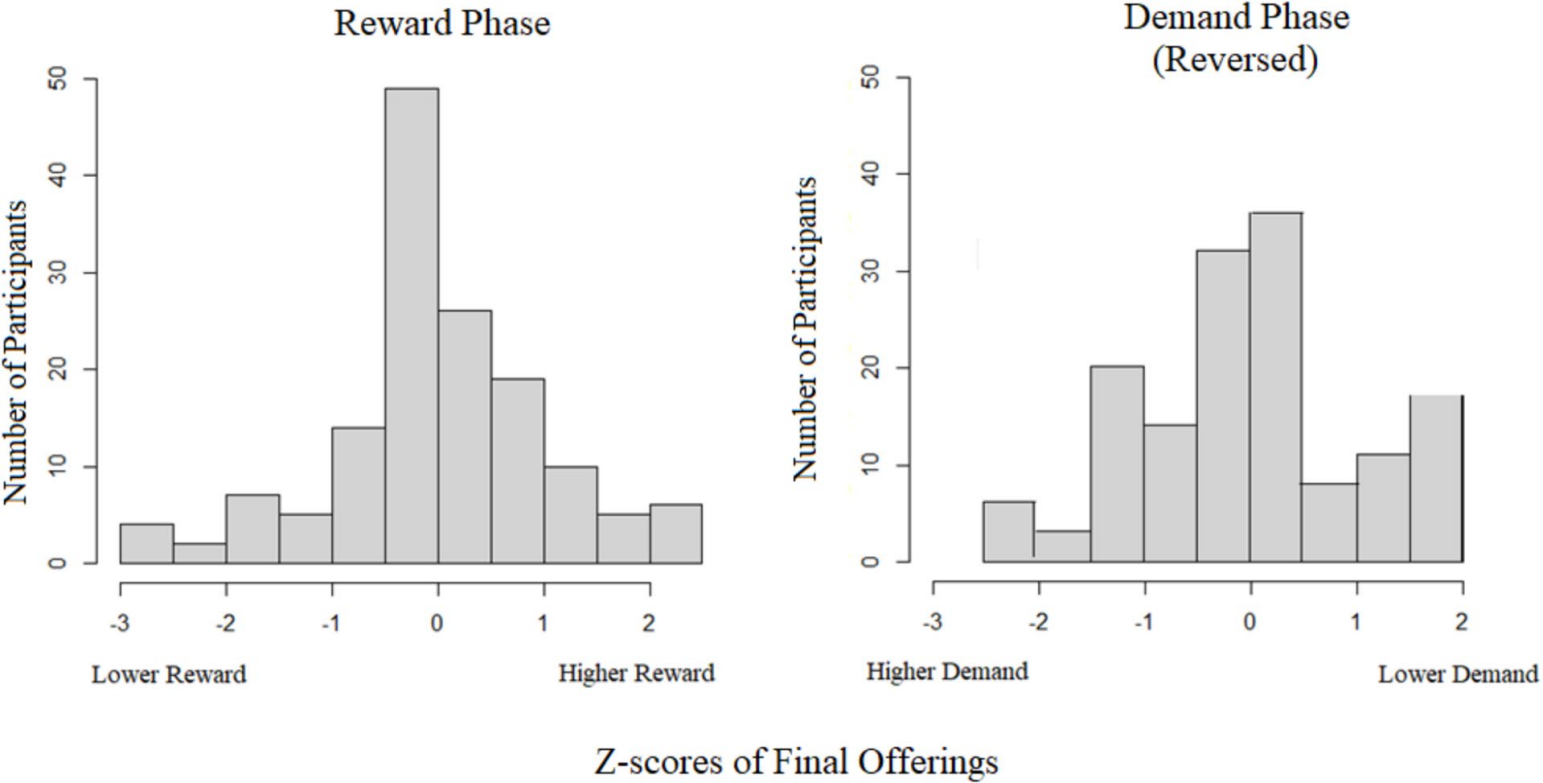
The figures display the distribution of final z-score offerings. The reward phase (**left**) is scaled from left to right as lower rewards (they kept choosing a task even as the offer lowered) to higher rewards (they chose the task with the highest reward offer). The demand phase (**right**) was reversed to match the biased direction as the reward and is scaled from left to right as higher demand (they kept choosing a task even as the demand level increased) to low demand (they chose the task with the lowest demand offer). The demand phase scale was reversed to facilitate a direct comparison of the two panels

The distribution of z-scores across both conditions shows that the reward phase had a wider range [−2.576, 2.138] than the demand phase [−1.883, 2.469] (Fig. 10). That is, while there seemed to be individual differences in how participants reacted in both phases, there may also be a general pattern of decisions made according to incentive type. The kurtosis of final answers was 0.571 for the reward phase and −0.17 for the demand phase. Extreme z-scores suggest that participants tend to consistently select one task throughout a given choice set, while scores that center around zero indicate that participants tend to sample both tasks within a given choice set. These latter choices were likely more heavily impacted by incentive type and less by the task options themselves. This is due to the adjustment of the secondary condition, in that choices throughout the experiment change which options are more rewarding or less demanding. The kurtosis results indicate that more participants were affected by the monetary amounts in the reward phase than the demand levels in the demand phase.

#### Questionnaire results

This study used two subjective scales: the Need for Cognition (NFC) scale (Cacioppo & Petty, 1982) and the reward responsiveness section of the Behavioral Activation System (BAS) scale (Carver & White, 1994). Cronbach’s alpha for the NFC consisted of 18 items (*α* = 0.969), and the BAS consisted of five items (*α* = 0.822). As expected, these results indicated high internal reliability. The results for the NFC scale (*M* = 2.83; *SD* = 0.56) indicated that participants were not particularly motivated for or against challenges. The results from the BAS scale (*M* = 4.40, *SD* = 0.51) suggested a high amount of sensitivity to reward among the participant sample.

One possibility is that the questionnaires included in the experiment would help account for some of the individual differences. However, Pearson’s correlation between the questionnaires and final z-scores of incentive phases did not indicate a significant relationship. The BAS score (*r* =.040, *p* =.628) and the NFC scores (*r* = -.007, *p* =.933) were not significant for the demand phase. The BAS score (*r* = -.056, *p* =.502) and the NFC scores (*r* = -.041, *p* =.627) were also not significant for the reward phase. This could be because this design was meant to compare how different tasks influence decisions under different effects of incentives. The NFC identifies individuals who seek out challenging pursuits, and the BAS scale considers those who are influenced by reward. This current design also adds to the motivation of different task types that may have a more confounding effect on decisions than those measured by these scales.

### Discussion

Experiment 2 investigated the influence of task type in two incentive phases to investigate whether decisions differed between different incentives and whether task type can further influence decision-making. Complimentary to the results found in Experiment 1, these results suggested that performance in a task cannot predict a task preference in that the Hybrid task was selected despite showing relatively lower performances when compared to the secondary tasks. Decisions across both incentive types revealed that there is within-subject validity in that people who make decisions favoring the lower-demand options tend to make decisions favoring high-rewarding options. However, there was a difference in incentive conditions in that more participants favored monetary-related options, whereas, in the demand condition, these preferences appeared to be influenced by task designs. Specifically, in the reward condition, more participants had final decision z-scores that indicated choices favoring incentive differences compared to the demand phase. These results further indicate that effort-based decision-making can be influenced by task designs as well as the incentives of demand and monetary rewards.

## General discussion

This study investigated action selection in cognitive effort-based decision-making. Theories of intrinsic motivation suggest that actions can be selected based on their utility for longer-term overarching goals (Aubret et al., 2023), the influence that performing an action has on a current stage of relieving boredom or fatigue (Agrawal et al., 2022), or simply because performing the action itself can be motivating despite not being related overtly to a long-term reward (Oudeyer, 2018). While the results of this study cannot completely rule out a current state-oriented hypothesis, these results do suggest that intrinsic motivation may be more strongly related to the action itself, rather than strictly how demanding the action is. Specifically, individual differences in effort investment appeared to be motivated by a myriad of factors such as the type of task being offered, performance-based factors, or external rewards such as money or points.

Experiment 1 used a 2 (demand level) × 3 (task type) design to investigate task decisions that were related to performance. Results suggested that about 20% of participants showed a demand-related pattern of decisions, 21% indicated a ranked-order task preference pattern, and exploratory analysis suggested an additional 24% may have been making decisions biased towards one of the three tasks. These results replicate past studies, which found that the majority of people (1) make decisions that are related to their performance levels, and (2) are primarily demand-avoidant (i.e., they choose tasks in which they perform better). However, there were still participants who indicated that their demand preferences actually biased them to select tasks they performed worse in, supporting theories that some people can be intrinsically motivated to seek out challenging options (Cacioppo & Petty, 1982; Pink, 2011; Ryan & Deci, 2000). These results suggest that performance and task types can influence participants in different ways. Experiment 2 used a within-subjects design to compare how different types of incentives can influence decisions. The results suggested that most participants were similarly influenced by fluctuations to task demand (as evoked by adding or taking away elements and response requirements to a task) and monetary amounts. Incentive conditions did differ slightly when it came to task preferences. Both tasks indicated that some participants were primarily motivated by the task options. However, more of these participants were more willing to forgo an easier task than to forgo a higher monetary reward. This could indicate that demand-oriented decisions may interact more with task-oriented decisions than monetary ones.

When comparing performance, there did not appear to be a strong relationship between performance and how strongly a participant preferred a particular task. These results suggest that demand levels or difficulty of a task may influence the willingness to select a task, and performance may then influence another aspect of decision-making, such as effort invested in completing the task after the task has been selected. This would complement theories such as task switching, which suggest that the decision to switch away from or select a new option may form its own demand (e.g., Monsell, 2003). This could explain why some participants persist with a given task option rather than an overall preference for a specific task. Together, these results suggest that task designs can reveal the various dimensions that decision makers use to make effort-based decisions. However, while these results emphasize individual differences in motivation for demand-related decisions, there is also a suggestion that these motivations may not be random. Instead, they suggest that how previous experiments defined demand and the weight they place on performance levels may be two of many factors that participants use when deciding to invest their cognitive resources, time, and effort into a given decision. The rest of this discussion is divided into three parts:

1. Viewing results through a resource-oriented lens,
2. Exploring aspects of the tasks that could have influenced decisions, and
3. Suggesting how these results support curiosity-driven and learning-oriented models of intrinsic motivation.

Resource-oriented decisions suggest that there is an inherent rationale in which decision-makers weigh the costs of performing an action against the benefits gained (e.g., Rangel et al., 2008; Shah & Oppenheimer, 2008). Traditionally, costs are associated with cognitive resource demands and benefits with some external reward such as money. However, recent theories suggest that motivations for internal rewards, such as those gained while satisfying curiosity or learning, may satisfy similar drives to external rewards (e.g., Mordirshanechi et al., 2024). Some models suggest that internal states can influence decisions by placing the decision-maker in an optimal state in which they seek out information or experiences that can further add to their current knowledge and skills while avoiding decisions that can lead to tasks that they cannot perform or are too easy (e.g., Agrawal et al., 2022). Other theories suggest that this motivation to seek out internally rewarding experiences may be related to the intrinsic motivation sought when decision-makers are in a sparse environment where external rewards may be hard to come by. The decision-maker must continue exploring their environment (Aubret et al., 2023). The participants in this study could have been making similar decisions among the different task types and demand levels offered. However, they may have used different decision aspects, such as performance, task type, last task selected, etc., because these aspects may affect various ways in which they can be engaged. Some may have been engaged by the components (i.e., motor or memory) involved in the task, others may have used familiarity with a task, and others may have used incentives, task difficulty, and performance levels to guide this gauge of internal engagement.

Unfortunately, this study did not find evidence that a particular task component was favored over another. When comparing motor to memory to hybrid tasks, preferences for these tasks appeared to be differently motivated (i.e., some were selected more or particularly avoided). It did not seem to matter if the participant was tasked with memory, motor, or a combination of the two components. However, preferences for these tasks were still seen across both experiments that were not overtly related to performance. A possible motivator for these results could be differences in previous experiences with component type. These tasks were novel to all participants, but participants had various experiences with past tasks that were motor-oriented or memory-oriented. The novelty of the task could have masked individual differences in performances on other memory- or motor-oriented task types. Another possible reason for these results is that there might be sex differences in preferences. Future research could include sex as an independent variable and directly test how it affects preferences. It is possible that preferences can be differently affected by task demand or task components based on sex.

However, the final motivation considered in this article is learning-oriented. The learning progress hypothesis theory suggests that people explore their environment and actions available to test and build upon an internal model they have of the world (Oudeyer, 2018). The author of this theory went on to suggest a curiosity-driven model of learning, which expands on this idea by suggesting that people use these new experiences to further their skill and knowledge to move the decision-maker to other areas (Oudeyer, 2018). Areas here are either literal in a given physical location or metaphorical in an area of an internal model, such as the decision-maker’s knowledge structure of a given skill or topic. Given that all these tasks were novel to the participant, it could be a fair strategy to stick to one option and continue rather than bouncing between options. Like task-switching, this theory suggests a cost in taking on a new task not experienced when sticking to a current task. However, this theory postulates that the cost is in the learning opportunity that sticking to one option can provide. Like state-dependent theories of engagement, learning-oriented theories could explain that participants use a current state, such as performance, to indicate that they may get better at it given enough time or that the task itself may not be worth the continued pursuit. Future studies could explore how increases in performance over time may be related to the decision to persist with a current task option over switching to another.

## Conclusion

Studies of motivation readily acknowledge that motivation can influence effort-based decision making, but they primarily differ on what is motivating. Intrinsic motivation is related to the internal reward gained through performing actions rather than the motivation for the benefit gained after performing the action. This study supports this view by providing empirical evidence that tasks can be motivated outside of monetary means. Other theories suggest that demand-related aspects of tasks may be a primary motivator in that people will value a task depending on how well they can perform it. This study challenges this viewpoint by providing more variety in the demand selection, suggesting that performance may not be the only dimension a decision-maker values tasks on. However, this study does fall short when it comes to offering a satisfying explanation for why one task was preferred over another. Performance, demand levels (as manipulated by the responses required of the participant), and component type (motor, memory, both) all provided strong evidence of influence. Further, performance was measured here using practice trials and did not account for changes in performance over time (i.e., learning). This limits the generalization for supporting learning-oriented models, and future directions could explore decisions and performance over time. In addition, it would be beneficial to separate the motivations driven by task-switching demands from learning-oriented ones. Overall, this study supports the idea that people can be motivated by performing an action outside of the external reward that could be gained or by how well they can perform the task. This further supports theories of intrinsic motivation, which state that people can be internally motivated to seek out experiences or actions.

## Supplementary Information

Below is the link to the electronic supplementary material.

Supplementary file1 (DOCX 578 KB)

## Data Availability

Data and analysis code are available via the Open Science Framework at: https://osf.io/657qr/?view_only=4ed67b50e5aa44e0b0e58b3e1fd06669 "Experiment 1 data and analysis code are available at https://osf.io/657qr/?view_only=4ed67b50e5aa44e0b0e58b3e1fd06669 Experiment 2 data and analysis code are available at https://osf.io/657qr/?view_only=4ed67b50e5aa44e0b0e58b3e1fd06669
