# Supplementary material for: Unraveling the influence of task designs and intrinsic motivation in effort-based decision-making
Source: Mem Cognit. 2025 Jul 8;54(1):200–15. doi: 10.3758/s13421-025-01745-6 (PMC12864360; doi:10.3758/s13421-025-01745-6)
Supplement: Supplementary file 1 — Supplementary file1 (DOCX 578 KB) [file 13421_2025_1745_MOESM1_ESM.docx]

**Appendix**

Experiment 1 used 12 different models based on the QTEST algorithm (Regenwetter et al., 2014). The first set of six models evaluated different response patterns based on participants’ performances in each task and level. These models were built with two specific patterns in mind: demand-avoidance and challenge-seeking. These were constructed to explore different decision strategies based on performance. There are three primary types: 1) those based on overall performance (Table A1), 2) on performance in low-demand tasks only (Table A2), and 3) those based on high-demand tasks only (Table A3). It is important to note that these models were built individually for each participant since they precisely predict preferences based on their performances.

**Overall Performance-Based Models*.***

These models did not consider differences among task types and instead weighed all six options by individual performance. Two models were built to capture two possible motivations: 1) those who sought to conserve effort (Demand Avoidant (DA)) and 2) those who instead sought out challenges (Challenge-Seeker (CS)). These models mirror each other, representing similar decision strategies. Model CS represents ideal responses from challenge-seeking participants in that these participants preferred an option based on how challenging that option is, as inferred by lower performance. Model DA represents ideal responses from demand-avoidant participants in that higher ease of demand (i.e., higher performance) can increase how likely that person was to choose that option. Suppose the participant found the two options equal. In that case, there would be no clear preference for one choice over the other—that is, each time that option was presented, the probability of that participant picking one over the other would not be above chance.

To elaborate, one participant had the following accuracy for the tasks (one was coded for low-demand options and two was coded for high-demand options): Memory (1 - .82/ 2 - .69), Motor (1 - .82/ 2 - .69), and Hybrid (1 - .88/ 2 - .51). The model demand-avoidant model would predict that when faced with two options, the participant preferred options they had previously had higher accuracy in. The order would be Hybrid 1. Memory.1, Motor.1, Motor.2, Memory.2, then Hybrid.2. On the other hand, the challenge-seeking model would predict that participants instead reversed this order of task preferences and selected options they previously had a lower accuracy in. If two options had similar accuracy, then the prediction would be equal chance between the two options (i.e., if a participant had .82 in both Memory.1 and Motor.1).


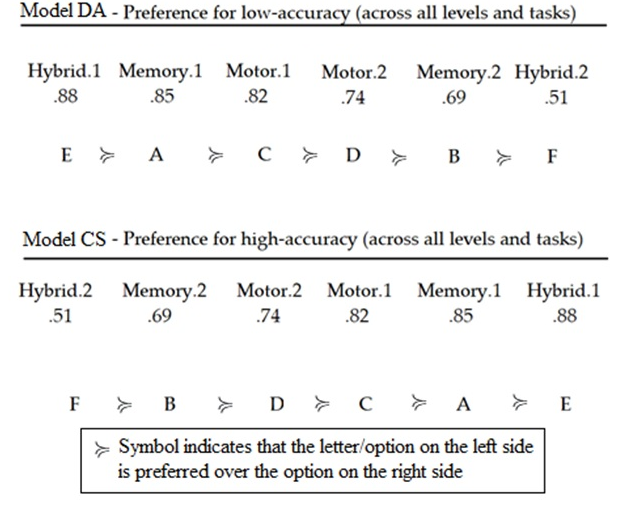


Table A1. Example of Models CS & DA built from a participant’s accuracy in the six options. Letters were assigned to each task (A: Memory.1; B: Memory.2; C: Motor.1, D: Motor.2; E: Hybrid.1; F: Hybrid.2).

**Low/High-Demand Performance-Based Models.**

Previous research has suggested that participants use heuristics to make decisions between options—that is, when given a choice, they will not only choose options based on demand levels, but the strategies participants use to make these decisions are also generalized (e.g., Cooper-Martin, 1994; Pizlo & Stefanov, 2013). In this case, the following four models were created to explore the possibility that participants’ preference for a task was based on one dimension of that task, specifically on their performance in either the low- or the high-demand version of a task.

***Low-Demand Performance-Based Models.***

The following two models only focus on performance in the low-demand task version (Table A2). These models predicted that preferences were based on the relationship along low-demand task options, not placing any weight on the high-demand versions. Model L: DA “Low: Demand-Avoidant” predicted that participants would prefer task options in which they performed better in the low-demand version. For instance, if the low-demand memory option had higher accuracy than the low-demand motor option, these participants preferred the memory task over the motor task every time these tasks were offered against each other. The second model, L: CS “Low: Challenge-Seeking,” is the same as the first model in that task preferences are based on the low-demand option. Instead of preferring options that they performed better than, these participants preferred options they found more challenging (i.e., have lower performance).

For example, using the previous participant data, these models predicted preferences based only on how the participant performed in the low-demand options (Memory.1 - .85, Motor.1 – 82, Hybrid -.88). The low-demand version of these models predicated preferences for tasks in the following order: Hybrid, Memory, then Motor). The high-demand version of these tasks still used these low-demand accuracy but suggested that preferences for these task options were in the reverse order: Motor, Memory, then Hybrid.


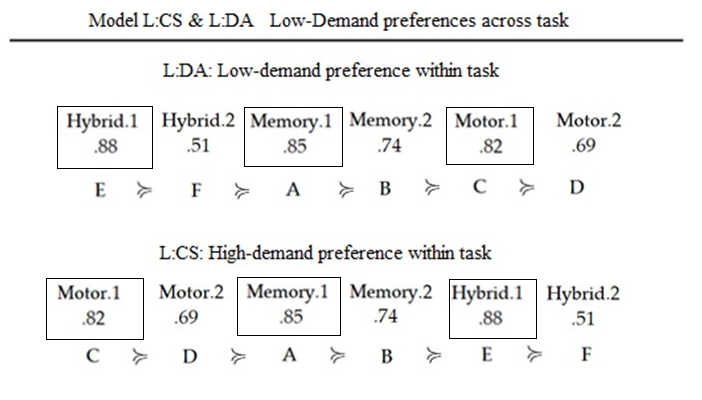


Table A2. Examples of Models Low-Demand: CS & Low-Demand: DA

***High-Demand Performance-Based Models.***

These final two models predicted that task preference was also based on a heuristic, but this heuristic is focused on performance in the high-demand task versions (Table A3). Model H: CS “High: Challenge-Seeking” predicted a bias towards the high-demanding option across tasks. For example, if the memory task had lower accuracy than the motor task, participant would prefer that task over the memory task. The fourth model, H: DA “High: Demand-Avoidant,” predicted that task preference was based on the ease of the high-demanding task without weighing performance in the low-demand task options.

Using the same participant example as before, these models predicted that performance in the high-demand options ordered preferences (Memory.2 - .74, Hybrid.2 - .55, Motor.2 - .69). The low-demand version of these models predicted the order of preference was as follows: Memory, Motor, then Hybrid. The high-demand version predicted the reverse order: Hybrid, Motor, then Memory.


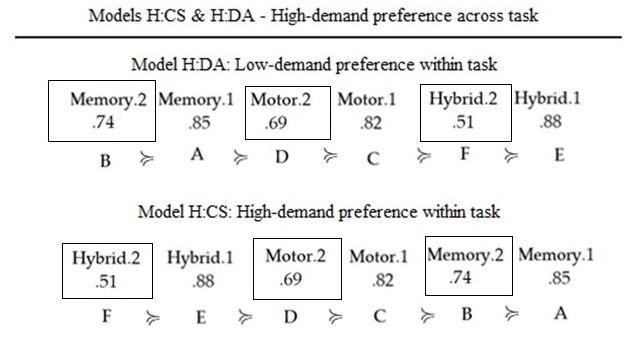


Table A3. Examples of Models High-Demand: CS & High-Demand: DA

**Task Models**.

The second set of six models tested whether task preferences could be ranked in order – regardless of performance (see Table A4). These models attempt 1) to investigate whether there was task-specific decision strategies and 2) to detangle whether these strategies were related to components specific to the tasks themselves. Rank ordering comparisons predict that there is a transitive property to task preferences. If a participant preferred task A over task B and task B over task C, they then preferred task A over task C. This transitive property defines a task preference as consistent across three tasks or whether decisions are chosen randomly. It is important to note that, unlike the previous demand-related models, these task models were the same for all participants.

For example, if a person preferred the memory task over the motor task and the motor task over the hybrid task, then they preferred the memory task every time they were offered it. These six models represented every arrangement of possible task preference combinations. Model WMH predicted that the memory task would be preferred over the Motor task, which would be preferred over the Hybrid task. Model WHM predicted this order would be Memory task over Hybrid task over Motor task. Model HWM: Hybrid task over Memory task over Motor task. Model HMW: Hybrid task over Motor task over Memory task. Model MWH: Motor task over Memory task over Hybrid task. Model MHW: Motor task over Hybrid task over Memory task. Brackets () indicate there is no prediction between these two options.


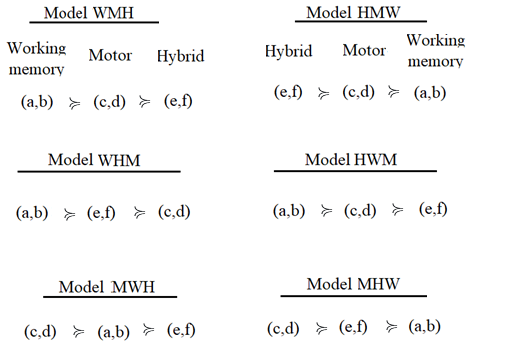


Table A4. Task Preference models for all participants
